# Corporate internal control, capacity utilization and total factor productivity

**DOI:** 10.1371/journal.pone.0318669

**Published:** 2025-02-11

**Authors:** Xiao Li

**Affiliations:** Systems and Industrial Engineering Technology Research Center, Zhongyuan University of Technology, Zhengzhou, Henan, China; Alexandru Ioan Cuza University: Universitatea Alexandru Ioan Cuza, ROMANIA

## Abstract

Based on Internal Control (IC) theory and Principal-agent theory, this study explores the impacts of IC on capacity utilization and total factor productivity, and the internal mechanism among them. The results show that effective IC improves total factor productivity and capacity utilization. Sufficient capacity utilization has a mediating effect for the impact of IC and total factor productivity. Heterogeneity discussion shows that with higher environmental uncertainty, effective IC has a more significant marginal effect on total factor productivity and capacity utilization, and sufficient capacity utilization has a greater mediating effect between IC and total factor productivity. Finally, it is suggested that regulators guide enterprises to strengthen IC construction, to improve capacity utilization and total factor productivity. Enterprises facilitate the mechanism that effective IC improves capacity utilization, and increases total factor productivity. This study enriches the literature on IC enabling corporate operation, and has practical significance for shaping competitive advantages.

## 1. Introduction

Institutions are a key factor affecting organizational performance. Corporate IC is an internal supervision and risk management mechanism. In 2008, China’s Ministry of Finance and other four ministries and commissions issued the “Basic Norms for Corporate Internal Control,” and IC system tends to develop, comprehensively. Based on IC theory, effective IC reasonably ensures business legal compliance, asset safety, authentic and integral financial statements and related information, facilitates operating efficiency and effectiveness, and supports development strategies. Total factor productivity means the additional output efficiency with given factor inputs [1], reflecting the overall efficiency of converting production factor inputs into outputs. It can be used to appraise macroeconomic quality and sustainability [2], as well as measure corporate operating efficiency and development quality [3]. For emerging economies, the improvement of total factor productivity is the key to shifting from high-speed growth to high-quality development [4]. So, does effective IC improve corporate total factor productivity?

Based on Principal-agent theory, agents allocate production capacity to the projects that are beneficial to own interests, but not conducive to corporate development, due to private motives, resulting in overcapacity and lower resource utilization efficiency [5]. Since the 1990s, overcapacity has plagued China’s economy [6]. Capacity utilization is the ratio of corporate actual output to theoretical maximum output [7], indicating whether an enterprise has overcapacity [8]. Effective IC mitigates agency risks at all levels within an enterprise, to achieve organizational objectives [9]. So, does effective IC resolve overcapacity, and improve capacity utilization? And capacity utilization is an important basis for explaining corporate behaviors [10], reflecting organizational efficiency. Further, is there a specific mechanism among IC, capacity utilization and total factor productivity?

In accordance with the above practical considerations, based on IC theory and Principal-agent theory, this study examines the impacts of IC on total factor productivity, and capacity utilization, and the internal mechanism among effective IC, capacity utilization and total factor productivity, to promote enterprises to strengthen IC effectiveness, and provide empirical evidence for improving capacity utilization, increasing total factor productivity, and achieving high-quality development. The remaining parts are organized as follows. Section 2 displays Literature review. Section 3 conducts Theoretical analysis, and research hypothesis. Section 4 presents Data source, variable definition and model setting-up. Section 5 shows Descriptive statistic and correlation. Section 6 carries out Model regression analysis. Section 7 renders Robustness test. Section 8 provides Heterogeneity discussion. And Section 9 draws conclusions and recommendations.

The contributions are as follows. Firstly, existing studies explored the mechanism among IC, financial mismatch and innovation performance [11], the moderating effect of environmental uncertainty while IC affected total factor productivity [12], and the effect of capacity utilization on total factor productivity [13]. However, few studies analyzed the inner link among IC, capacity utilization, and total factor productivity. This study reveals the mediating effect of capacity utilization for the impact of IC on total factor productivity, providing a beneficial reference for constructing a long-term mechanism to resolve overcapacity and increase total factor productivity. Secondly, given that environmental uncertainty is an important factor affecting operational decisions [12, 14], based on differentiated environmental uncertainty, this study explores the heterogeneous effects of IC on total factor productivity, and capacity utilization, respectively, enriching the relevant literature on IC economic effects, and expanding the research boundary between IC and operational efficiency. Thirdly, in compliance with differentiated environmental uncertainty, this study elucidates the heterogeneous mechanism that IC improves capacity utilization, and increases total factor productivity, which is of significance to achieve efficiency improvement and high-quality development.

## 2. Literature review

As for IC economic effect, existing studies have not reached a consensus. For instance, Bargeron et al. (2010) [15] argued that strict IC mechanism was not conducive to innovation businesses. In contrast, Johnstone et al. (2011) [16] believed that IC optimized corporate internal process through contracts, reasonably distributed stakeholders’ rights and responsibilities, supervised and balanced power, and improved corporate governance. Higher-quality IC enhances corporate stock liquidity [17]. Additionally, existing research has explored capacity utilization. In emerging economies undergoing transition, corporate operation relies more on the government’s industrial policies, and there is a serious phenomenon of “easy to expand, but hard to retreat” in capacity expansion, resulting in relatively common overcapacity [18], consequently, production potential has not been fully utilized, and the actual output is lower than potential capacity, which reduces capacity utilization efficiency. Although resolving overcapacity is a macro proposition, production capacity arises from micro corporate behaviors [19].

Moreover, existing studies have explored the relationship between capacity utilization and total factor productivity. As a competitiveness index that deserves attention during the new development phase, total factor productivity measures corporate technological progress, and reveals hard-to-measure factors such as management skills, institutional innovation and growth potential [20, 21]. However, in China, current capacity utilization cannot promote total factor productivity in industrial development, which is considered as the “capacity utilization paradox” [13]. To a larger extent, capacity utilization is determined by enterprises based on operating conditions, while as a comprehensive reflection of production efficiency, total factor productivity is difficult to be directly determined by managers. In the long run, lower capacity utilization and production factors’ output efficiency is not conducive to optimizing and upgrading industrial structure, which increases economic risks [22], and affects corporate asset returns adversely [23].

According to existing studies, scholars have explored IC economic effects, but no consensus has been reached. And existing studies have explored capacity utilization in emerging economies undergoing transition, suggesting widespread overcapacity [18], and the “capacity utilization paradox” [13]. Long-term low capacity utilization increases economic risks [22]. To resolve overcapacity, it is necessary to identify its causes of overcapacity, and the factors leading to irrational expansion at corporate level. However, few studies have comprehensively considered the internal logic among IC, capacity utilization, and total factor productivity. Therefore, this endows an opportunity for elucidating the impacts of effective IC on total factor productivity and capacity utilization, as well as the mechanism among them. This not only enriches the relevant literature on IC economic effects, but also offers practical insights for emerging economies to resolve overcapacity and increase total factor productivity.

## 3. Theoretical analysis and research hypothesis

### 3.1 IC and total factor productivity

As micro entities in the market economy, whether enterprises acquire growth in total factor productivity directly affects whether the macro-economy achieves high-quality development. Based on IC theory, effective IC monitors operational decision-making and implementation through the controls at the overall level such as organizational structure, decision-making and execution mechanisms, as well as those at the business level such as control procedures and methods, to inhibit agents’ opportunism, and reduce agency costs. As an essential governance mechanism, effective IC runs through corporate decision-making, implementation and supervision [24]. IC involves five elements, i.e., internal environment, risk assessment, control activities, information and communication, and internal supervision.

The internal environment is the basis for IC implementation, constructing a normative governance structure and procedural rules, defining decision-making, implementation, supervision and other responsibilities, and establishing scientific division mechanisms. Based on checks and balances, a good internal environment creates mutual restrictions and supervision in organization settings, rights and responsibilities’ distribution, and business processes, thereby reducing agency risks, and improving operational efficiency [11]. With risk assessment and control activities, enterprises identify and systematically analyze risks timely, develop appropriate response strategies, and implement solutions to mitigate risks, to ensure smooth operation, and increase total factor productivity. And efficient information and communication are conducive to enhancing various departments’ capabilities to discover and address risks, improve information transmission efficiency, and attenuate information asymmetry [25], to reduce principal-agent costs, and enhance operational efficiency. In addition, internal supervision creates power constraints between owners and operators, weakens managerial opportunism to a great extent, encourages managers to better perceive and anticipate changes in external economic conditions, guard against systemic risks, and improve job performance. Effective IC alleviates agency conflicts, improves production and operational efficiency [26], promotes corporate development quality, efficiency and momentum changes, and increases total factor productivity.

Based on the above analyses, the following research hypothesis is proposed.

**Hypothesis 1.** Effective IC increases total factor productivity significantly.

### 3.2 IC and capacity utilization

Based on Principal-agent theory, asymmetric and incomplete information may lead to “investment surge,” resulting in overcapacity [27]. Capacity utilization efficiency reflects corporate actual capacity utilization. Lower capacity utilization means that enterprises hold a substantial amount of idle capital assets, including machinery and equipment. Enterprises with stronger IC are more likely to utilize derivatives than those with weaker IC [28]. Based on IC theory, a favorable internal environment is conducive to setting operational objectives [29], ensuring the all-round controls of the board of directors and the management over enterprises [30], preventing potential risks, and improving the abilities to identify and cope with risks. Effective IC fosters the identification of internal and external environmental risk factors and timely risk assessment, enhances managers’ sensitivities to the market and projects, and mitigates the adverse impact of uncertainties on capacity utilization.

Control activities optimize corporate operation, allocate resources more efficiently [21], reduce resource waste, so that the “prior” production capacity is fully utilized, mitigating potential adverse impacts on capacity utilization. Information and communication ensure that information is communicated among stakeholders, guide market demand expectations, and effectively improve supply and demand matching. Good information transparency enables enterprises to grasp market dynamic changes, access demand information more easily, match target customers accurately, reduce information search costs, and improve transaction efficiency [31]. Effective IC improves accounting information quality, ensures financial statements to reflect financial status, operating results, and cash flow objectively, thereby evaluating managerial efforts more fairly [32], creating a sound supervision mechanism, alleviating opportunism, and encouraging the management to make reasonable operational arrangements. Moreover, effective IC motivates employees’ enthusiasm for production, with reasonable incentive mechanisms, enhancing capacity utilization.

Based on the above analyses, the following research hypothesis is proposed.

**Hypothesis 2.** Effective IC enhances capacity utilization significantly.

### 3.3 IC, capacity utilization, and total factor productivity

Based on IC theory, with preventive and discovery controls, effective IC detects human resource, management, financial, safety, and environmental risks promptly, and mitigates operational risks within acceptable limits, enhances resource allocation efficiency, improves total factor productivity and capacity utilization. At the micro level, capacity utilization is more a cause than a result for total factor productivity changes [33]. Capacity utilization improvement reduces extensive factor utilization and resource waste, lowers the energy consumption per unit of product, realizes intensive factor utilization, narrows the deviation between the “pre-production” capacity and “post-digested” operational capacity, alleviates business risks, and improves output efficiency.

Indeed, the dynamic changes in capacity utilization partly explain those in total factor productivity [34]. Capacity utilization improvement helps increase total factor productivity [33]. When capacity utilization is higher, and maintains steady growth, the utilization of existing factors meets expectations. Higher capacity utilization implies greater output efficiency, when other things are equal. Improving capacity utilization is an essential measure to increase total factor productivity. Further, considering Hypothesis 2 that effective IC enhances capacity utilization, this study argues that enterprises implement the controls at the overall level such as organizational structure, decision-making and execution mechanism, as well as those at business level such as control procedures and methods, supervising operational decisions and behaviors, optimize capacity utilization, and thereby increase total factor productivity.

Based on the above analyses, the following research hypothesis is proposed.

**Hypothesis 3.** Corporate capacity utilization has a significant mediating effect for the impact of IC on total factor productivity.

## 4. Data source, variable definition, and model setting-up

### 4.1 Data source

In 2012, China Securities Regulatory Commission issued the revised “Guidelines on Listed Companies’ Industry Classification.” To facilitate industry classification, the listed enterprises in China’s capital markets from 2012 to 2022 are taken as the sample. Where, corporate basic characteristic data are from Wind financial terminal; IC data are from “Dibo · IC Index of Listed Companies in China,” issued by Shenzhen Dibo Enterprise Risk Management Technology Co., Ltd.; and other data are derived from the following calculations or descriptions. To ensure integrity and reliability, the annual observations with missing data are excluded. In view of the particularity of financial statements in the financial industry [35], those in the financial industry are excluded. And those treated by ST, or *ST are excluded. Finally, the data from 3275 enterprises are considered as valid observations. In addition, the bidirectional 1% Winsorization is applied to continuous variables, to reduce outliers’ adverse effects.

### 4.2 Variable definition

Table 1 shows the variables’ names and descriptions.

**Table 1:** Variable name and description.

Nature	Symbol	Name	Calculation method
Explained variable	TFP_LP	Total factor productivity	Acquired from LP method
Explanatory variable	IC	IC effectiveness	Dibo · IC Index
Mediating variable	CAUT	Capacity utilization	Acquired from SFA method
Control variable	LEV	Asset-liability ratio	Total liabilities/total assets
	TAT	Total assets turnover	Operating income/average total assets at the beginning and end
	ROA	Return on total assets	Net profits/average total assets at the beginning and end
	TQ	Corporate growth	Corporate market value/total assets
	R&D	R&D investment	The percentage of R&D investment to operating income
	ShrZ	Ownership concentration	Shareholdings of the largest shareholder/those of the second largest shareholder
	ManaHold	Management shareholding	The ratio of the management shareholdings to general capital
	LnSALARY	Executive compensation	The natural logarithm of top three executive salaries
	LnASSET	Corporate scale	The natural logarithm of total assets
	SOE	Property attribute	Dummy variable, 1 for state-owned enterprises; otherwise, 0
	YEAR	Year	Annual effect
	IND	Industry	Industry effect; dummy variables set according to the “Guidelines on Listed Companies’ Industry Classification”
	ε		Random disturbance term

#### 4.2.1 Explained variable

Comprehensively, corporate total factor productivity reflects production efficiency. Mainly, the methods for measuring total factor productivity include parametric, non-parametric and semi-parametric methods. After comparing total factor productivity in China, Lu and Lian (2012) [36] found that the semi-parametric method alleviated the endogeneity and sample selection bias in traditional measurement methods. And compared to current investments, Levinsohn-Petrin (LP) method is more sensitive to total factor productivity and less missing [37]. Therefore, with reference to Levinsohn and Petrin (2003) [38], Lu and Lian (2012) [36], Chen et al. (2020) [37], the following Model 1 is constructed, and LP method is adopted to measure total factor productivity.

**Model 1.**

$$LnY_{i,t}=\alpha LnL_{i,t}+f(LnK_{i,t},LnM_{i,t})+\varepsilon_{i,t}$$ (1)

In Model 1, LnY_i,t_ represents corporate total operating output, which is the natural logarithm of operating income of enterprise ***i*** in year ***t***; LnL_i,t_ represents labor input, expressed by the natural logarithm of the total number of employees; *f*(LnK_i,t_,LnM_i,t_) is a function of the capital stock LnK_i,t_, and the mediating input LnM_i,t_. LnK_i,t_ is the natural logarithm of net fixed assets. For M_i,t_, Eq (2) shows the calculation. MA_1_ ~ MA_4_ represents operating costs, sales expenses, financial expenses, administrative expenses; MB_1_ ~ MB_2_ is the depreciation and amortization, cash paid to and for employees, respectively. In accordance with Model 1, the estimated coefficients on LnL_i,t_, and LnK_i,t_ are substituted into Eq (3), to estimate total factor productivity based on LP method (TFP_LP).


$$M_{i,t}=\sum_{n=1}^{4}MA_{n}–\sum_{n=1}^{2}MB_{n}$$ (2)

$$TFP\_LP_{i,t}=LnY_{i,t}-\hat{\alpha }LnL_{i,t}-\hat{\psi }LnK_{i,t}$$ (3)

#### 4.2.2 Explanatory variable

In 2011, in line with the achievement of IC objectives, involving compliance, reporting, asset safety, operation and strategy, as an independent third party, Shenzhen Dibo Enterprise Risk Management Technology Co., Ltd. issued the “Dibo · IC Index of Listed Companies in China.” This index evaluates corporate IC effectiveness, reflecting IC level and risk management capacity [11]. Therefore, this index is adopted, to measure corporate IC effectiveness. The greater the index, the more effective the IC.

#### 4.2.3 Mediating variable

For corporate capacity utilization (CAUT), the commonly used measurements involve ratio, peak, cost function, data envelopment analysis, and stochastic frontier production function methods [23]. At corporate level, stochastic frontier analysis (SFA) is recognized by existing studies, believing that it is more direct, and aligned with the purpose of evaluating capacity utilization, to determine frontiers from a production perspective [7, 39], which not only considers production factor utilization and technological progress, but also effectively excludes random errors [19]. Thereby, with reference to Li et al. (2017) [7], and Du et al. (2022) [39], the prime operating income, total assets and the number of employees are adopted to construct a stochastic frontier. With SFA method, the ratio of actual output to frontier output is regarded as CAUT.

#### 4.2.4 Control variable

With reference to Li and Zhao (2022) [11], Asset-liability ratio, Total assets turnover, Return on total assets, Corporate growth, R&D investment, Ownership concentration, Management shareholding, Executive compensation, Corporate scale, and Property attribute are taken as the control variables, to examine the possible effects on capacity utilization, and total factor productivity. Also, annual and industry effects are considered in regression analyses.

### 4.3 Model *s*etting-up

With reference to Li and Zhao (2022) [11], and Zhang et al. (2024) [40], the following Models 2 to 4 are constructed, and fixed-effects analyses are conducted, to examine Hypotheses 1 to 3 above. Fixed-effects models have certain information advantages, mitigating the interference of unobservable variables that do not change with time. Among the control variables, LEV, TAT, ROA, TQ, R&D, ManaHold, LnSALARY, and LnASSET are taken as first-order lags in regression analyses, to mitigate the endogeneity caused by reverse causality. Moreover, to avoid excessive dimensional differences, in Models 2 and 4, the explained variable is the natural logarithm of TFP_LP, and expresses as LnTFP_LP; from Models 2 to 4, IC is the normalized value that the “Dibo · IC Index” is divided by 1000.

Model 2.


$$\begin{array}{l} LnTFP\_LP_{i,t}=\beta_{0}+\beta_{1}IC_{i,t}+\beta_{2}LEV_{i,t‐1}+\beta_{3}TAT_{i,t‐1}+\beta_{4}ROA_{i,t‐1}+\beta_{5}TQ_{i,t‐1}+\beta_{6}R\&D_{i,t‐1}+\\ \;\beta_{7}ShrZ_{i,t}+\beta_{8}ManaHold_{i,t‐1}+\beta_{9}LnSALARY_{i,t‐1}+\beta_{10}LnASSET_{i,t‐1}+\\ \;+\beta_{11}SOE_{i,t}+\beta_{12}\sum_{t}YEAR+\beta_{13}\sum_{t}IND+\varepsilon_{i,t} \end{array}$$ (4)

Model 3.


$$\begin{array}{l} CAUT_{i,t}=\theta_{0}+\theta_{1}IC_{i,t}+\theta_{2}LEV_{i,t‐1}+\theta_{3}TAT_{i,t‐1}+\theta_{4}ROA_{i,t‐1}+\theta_{5}TQ_{i,t‐1}+\theta_{6}R\&D_{i,t‐1}+\\ \;\theta_{7}ShrZ_{i,t}+\theta_{8}ManaHold_{i,t‐1}+\theta_{9}LnSALARY_{i,t‐1}+\theta_{10}LnASSET_{i,t‐1}+\\ \;+\theta_{11}SOE_{i,t}+\theta_{12}\sum_{t}YEAR+\theta_{13}\sum_{t}IND+\varepsilon_{i,t} \end{array}$$ (5)

**Model 4.**

$$\begin{array}{l} LnTFP\_LP_{i,t}=\delta_{0}+\delta_{1}IC_{i,t}+\delta_{2}CAUT_{i,t}+\delta_{3}LEV_{i,t‐1}+\delta_{4}TAT_{i,t‐1}+\delta_{5}ROA_{i,t‐1}+\delta_{6}TQ_{i,t‐1}+\\ \;\delta_{7}R\&D_{i,t‐1}+\delta_{8}ShrZ_{i,t}+\delta_{9}ManaHold_{i,t‐1}+\delta_{10}LnSALARY_{i,t‐1}+\\ \;\delta_{11}LnASSET_{i,t‐1}+\delta_{12}SOE_{i,t}+\delta_{13}\sum_{t}YEAR+\\ \;\delta_{14}\sum_{t}IND+\varepsilon_{i,t} \end{array}$$ (6)

**Fig 1:**
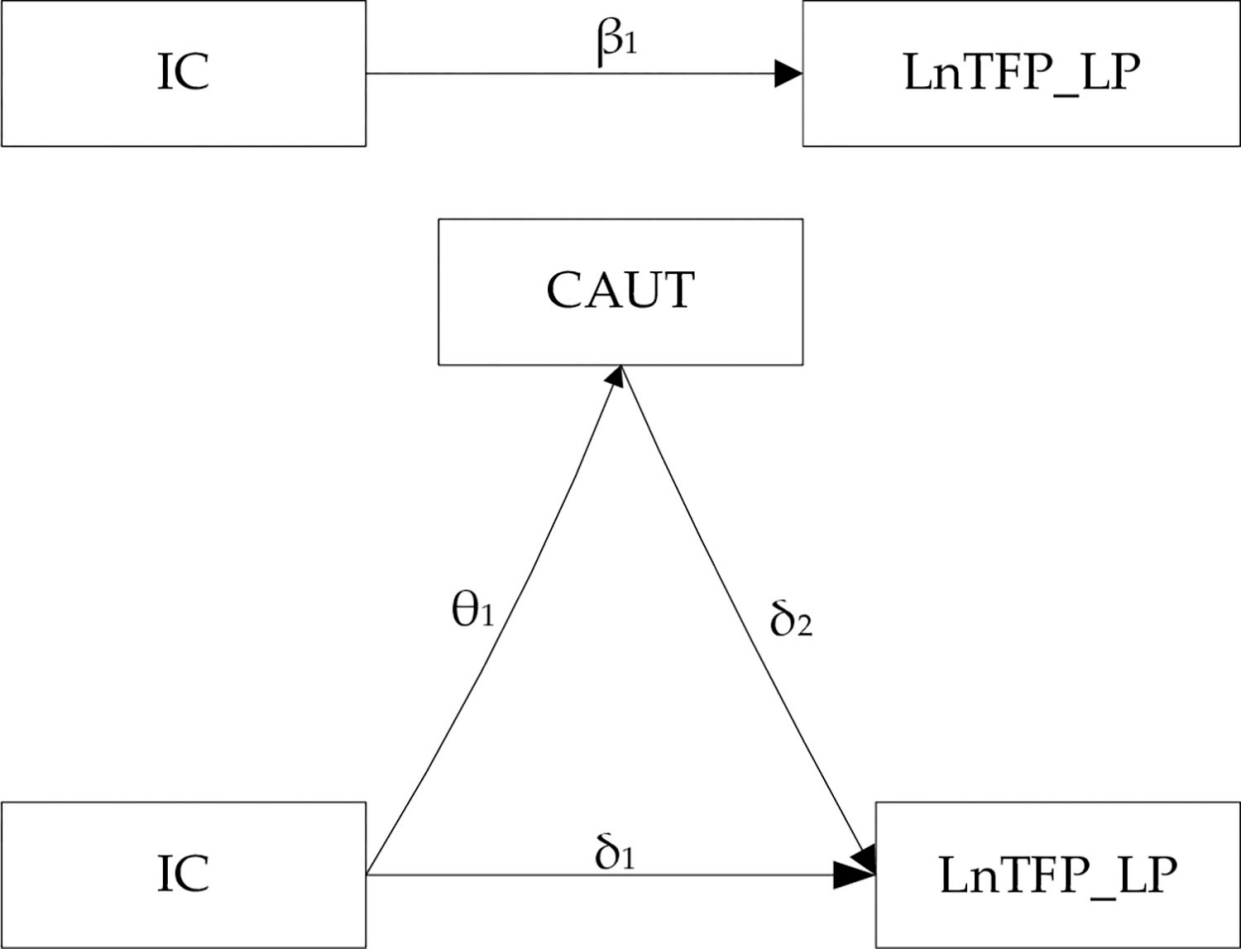
Mediation framework diagram.

With reference to Li and Zhao (2022) [11], briefly, Fig 1 describes the mediation effect framework. In Model 2, the coefficient β_1_ represents the total effect of IC on total factor productivity. In Model 3, θ_1_ represents the effect of IC on capacity utilization. In Model 4, δ_1_ is the direct effect of IC on total factor productivity, and δ_2_ is the effect of capacity utilization on total factor productivity. If δ_1_ is lower than β_1_, it indicates that capacity utilization has a significant mediating effect for the impact of IC on total factor productivity, meaning that effective IC improves capacity utilization, and thereby increases total factor productivity.

## 5. Descriptive statistic and correlation

### 5.1 Descriptive statistics

Table 2 reports the descriptive statistics. For the explained variable, the mean (standard deviation) of LnTFP_LP is 8.989 (1.012). Among enterprises, larger differences exist in total factor productivity, overall, which needs to be improved. For the explanatory variable, the maximum (minimum) of IC is 0.799 (0.000), and the mean is 0.641. In general, IC effectiveness is good, but there are substantial differences. For the mediating variable, the mean of CAUT is 67.81%, lower than the desirable capacity utilization, i.e., 75% [23]. Moreover, higher heterogeneity exists in capacity utilization, and overcapacity reduces resource allocation efficiency.

**Table 2:** Descriptive statistics.

Variable	Mean	Median	Maximum	Minimum	Deviation	Observations
LnTFP_LP	8.989	8.887	11.780	6.960	1.012	23486
IC	0.641	0.661	0.799	0.000	0.112	23486
CAUT	67.814	68.458	84.953	43.823	7.860	23486
LEV	39.899	39.007	85.418	5.676	19.152	23486
TAT	0.647	0.562	2.427	0.110	0.394	23486
ROA	4.365	4.144	22.780	-20.021	6.268	23486
TQ	2.092	1.688	8.020	0.854	1.268	23486
R&D	5.068	3.820	28.150	0.030	4.984	23486
ShrZ	7.480	3.408	72.000	1.003	11.332	23486
ManaHold	15.871	3.914	68.077	0.000	20.049	23486
LnSALARY	5.360	5.331	7.267	3.741	0.678	23486
LnASSET	22.164	21.998	25.708	20.111	1.170	23486
SOE	0.276	0.000	1.000	0.000	0.447	23486

In the control variables, the maxima (minima) of LEV, TAT, ROA, TQ, R&D, ManaHold, LnSALARY, and LnASSET are 85.42% (5.68%), 2.427 (0.110), 22.78% (-20.02%), 8.020 (0.854), 28.15% (0.03%), 68.08% (0.00%), 7.267 (3.741), and 25.708 (20.111). Debt pressure, asset turnover and returns, corporate growth, R&D input, management shareholding, executive compensation, and asset scale exhibit greater heterogeneity. On average, the largest shareholder holds a higher proportion of shares, and state-owned enterprises account for 27.60%. Overall, the sample is well differentiated, providing a beneficial basis for regression analyses.

### 5.2 Correlations

Table 3 reports the pairwise correlations. In Models 2 and 4, IC is positively and significantly correlated with LnTFP_LP (0.153, *p*< 0.01). In Model 3, IC is positively and significantly correlated with CAUT (0.123, *p*< 0.01). The more effective the IC, the higher the total factor productivity capacity utilization, providing a reference for enhancing total factor productivity, and capacity utilization. In Model 4, CAUT is positively and significantly correlated with LnTFP_LP (0.518, *p*< 0.01). An improvement in capacity utilization helps to raise total factor productivity.

**Table 3:** Pairwise correlations.

Variable	LnTFP_LP	IC	CAUT	LEV	TAT	ROA	TQ	R&D	ShrZ	ManaHold	LnSALARY	LnASSET	SOE	VIF
LnTFP_LP	1.000													
IC	0.153***	1.000												1.150
CAUT	0.518***	0.123***	1.000											2.820
LEV	0.502***	-0.079***	0.154***	1.000										1.850
TAT	0.568***	0.153***	0.785***	0.206***	1.000									2.730
ROA	0.129***	0.327***	0.213***	-0.341***	0.192***	1.000								1.590
TQ	-0.252***	-0.003	-0.005	-0.281***	-0.033***	0.241***	1.000							1.300
R&D	-0.336***	-0.020***	-0.324***	-0.309***	-0.309***	-0.030***	0.255***	1.000						1.400
ShrZ	0.128***	-0.009	0.036***	0.117***	0.067***	-0.040***	-0.071***	-0.140***	1.000					1.120
ManaHold	-0.299***	0.068***	-0.021***	-0.292***	-0.075***	0.150***	0.026***	0.196***	-0.209***	1.000				1.440
LnSALARY	0.434***	0.083***	0.137***	0.105***	0.142***	0.178***	0.001	0.087***	-0.089***	-0.109***	1.000			1.430
LnASSET	0.830***	0.091***	0.026***	0.511***	0.120***	0.024***	-0.318***	-0.252***	0.137***	-0.366***	0.416***	1.000		2.270
SOE	0.291***	-0.010	0.015**	0.276***	0.063***	-0.103***	-0.127***	-0.203***	0.285***	-0.455***	-0.005	0.359***	1.000	1.430

Note: *** Significant at 1%

** Significant at 5%

* Significant at 10%.

For the control variables, in Models 2 and 4, LEV (0.502), TAT (0.568), ROA (0.129), ShrZ (0.128), LnSALARY (0.434), LnASSET (0.830), and SOE (0.291) are positively and significantly correlated with LnTFP_LP (*p*< 0.01). And TQ (-0.252), R&D (-0.336), and ManaHold (-0.299) are negatively and significantly correlated with LnTFP_LP (*p*< 0.01). In Model 3, LEV (0.154), TAT (0.785), ROA (0.213), ShrZ (0.036), LnSALARY (0.137), and LnASSET (0.026) are positively and significantly correlated with CAUT (*p*< 0.01); as is SOE (0.015, *p*< 0.05). And R&D (-0.324), ManaHold (-0.021) are negatively and significantly correlated with CAUT (*p*< 0.01). From Models 2 to 4, the correlations support the validity.

In addition, the maximum correlation between the control variables is 0.511, existing between LEV and LnASSET, below the threshold of 0.800; and in the last column, the maximum variance inflation factor is 2.82, indicating that there is no serious multicollinearity, and providing a reliable guarantee for subsequent regression.

## 6. Model regression analysis

Without considering other factors affecting the explained variables, the descriptive statistics and correlations serve as the preliminary results. From Models 2 to 4, Table 4 reports the results with fixed-effects regression, controlling for the fixed effects of firms and years.

**Table 4:** Regression results for Models 2 to 4.

Variable	(1)	(2)	(3)
	Model 2	Model 3	Model 4
	Coef. (S.E.)	Coef. (S.E.)	Coef. (S.E.)
Intercept	-2.775*** (0.296)	8.321*** (0.381)	-6.865*** (0.234)
IC	0.522*** (0.032)	0.459*** (0.046)	0.297*** (0.021)
CAUT			0.491*** (0.009)
L.LEV	0.102*** (0.039)	0.295*** (0.061)	-0.043 (0.027)
L.TAT	0.701*** (0.023)	0.819*** (0.033)	0.298*** (0.020)
L.ROA	0.327*** (0.075)	0.024 (0.110)	0.316*** (0.051)
L.TQ	0.035*** (0.003)	0.003 (0.005)	0.033*** (0.003)
L.R&D	-0.111*** (0.018)	-0.209*** (0.026)	-0.008 (0.012)
ShrZ	-0.021 (0.040)	0.087 (0.055)	-0.064** (0.029)
L.ManaHold	0.010 (0.042)	0.001 (0.060)	0.009 (0.032)
L.LnSALARY	0.006 (0.009)	-0.001 (0.013)	0.006 (0.006)
L.LnASSET	0.495*** (0.013)	-0.103*** (0.017)	0.545*** (0.010)
SOE	-0.050** (0.023)	-0.110*** (0.033)	0.004 (0.015)
YEAR/IND/Firm	YES	YES	YES
# of obs.	18727	18727	18727
Within_R^2^	0.636	0.245	0.814
F_Value	746.83***	138.86***	1826.86***

Note: *** Significant at 1%

** Significant at 5%

* Significant at 10%.

() denotes robust standard errors clustered at corporate level.

### 6.1 Analyses of Model 2’s regression results

In column 1, the coefficient on IC is positive and significant (0.522, *p*< 0.01), implying that effective IC contributes to increasing total factor productivity, which tends to be consistent with the research by Wang et al. (2023) [12]. In an economic sense, for each one standard deviation increase in IC (0.112), the average increase in the explained variable (LnTFP_LP) is equivalent to 5.78% of the sample standard deviation (i.e., 0.522×0.112/1.012). Effective IC optimizes operation processes, reasonably distributes stakeholders’ rights and responsibilities, ensures checks and balances on power, alleviates agency problems [16], and improves operation efficiency [26]. Moreover, effective IC improves performance indicators’ information content, to better reflect managers’ efforts [41], and correct operation decisions in line with managers’ preferences rather than stakeholders’ interests. Continuously, enterprises strengthen IC construction, promote operational quality, efficiency, power changes, and increase total factor productivity. Hypothesis 1 above is verified.

For the control variables, the coefficients on L.LEV, L.TAT, and L.LnASSET are positive and significant (0.102, *p*< 0.01; 0.701, *p*< 0.01; 0.495, *p*< 0.01). Good creditor governance, asset turnover and scale effect enhance total factor productivity. As are those on L.ROA, and L.TQ (0.327, *p*< 0.01; 0.035, *p*< 0.01). Good profitability and growth enable enterprises to respond quickly to changes in external environments, and increase total factor productivity. However, those on L.R&D and SOE are negative and significant (-0.111, *p*< 0.01; -0.050, *p*< 0.05). Possibly, R&D input has not been fully converted into actual productivity, and the transformation of scientific and technological achievements needs to be further improved. Due to political connections, to a certain extent, state-owned enterprises obtain more policy support, tax incentives and credit resources, etc., weakening the perception of external competitive pressure, and lacking the internal motivation to increase total factor productivity.

### 6.2 Analyses of Model 3’s regression results

In column 2, that on IC is positive and significant (0.459, *p*< 0.01), implying that effective IC promotes capacity utilization. Hypothesis 2 is verified. This conclusion is of greater economic significance. For each one standard deviation increase in IC, corporate capacity utilization increases by 7.58% on average (0.459×0.112/0.678). Effective IC mitigates internal and external information asymmetry, enhances decision-making efficiency, ensures sustainable operation, and alleviates overcapacity effectually. Effective IC improves capital allocation [29], promotes enterprises to utilize existing resources and capacities, encourages managers to make decisions in line with corporate interests and needs, allocates resources reasonably to projects aligned with stakeholders’ interests, and thus improves capacity utilization.

For the control variables, those on L.LEV, and L.TAT are positive and significant (0.295, *p*< 0.01; 0.819, *p*< 0.01). Good creditor governance and asset turnover motivate enterprises to fully utilize operating resources. However, that on L.R&D is negative and significant (-0.209, *p*< 0.01). Perhaps, due to insufficient patent industrialization, core components, system integration software, and high-end equipment have not achieved independent innovation, affecting existing capacity utilization adversely. And as are those on L.LnASSET and SOE (-0.103, *p*< 0.01; -0.110, *p*< 0.01). Driven by internal expansion, large enterprises make excessive investments, leading to overcapacity. In state-owned enterprises, the lower capacity utilization may result from the resource mismatch caused policy burden [42], or from blind waste due to soft budget constraints.

### 6.3 Analyses of Model 4’s regression results

In column 3, that on IC is positive and significant (0.297, *p*< 0.01). Effective IC improves operational efficiency, and increases total factor productivity, to achieve long-term development strategies. And as is that on CAUT (0.491, *p*< 0.01). All else being equal, higher capacity utilization means greater output, different from the view held by Feng (2017) [13], regarding the “capacity utilization paradox.” Capital allocation is an important factor affecting total factor productivity [43]. With sufficient capacity utilization, the utilization of existing equipment has met expectations, reducing large-scale overcapacities, and promoting total factor productivity. Further, considering those on IC from Models 2 to 4, and that on CAUT in Model 4, this study argues that sufficient capacity utilization has a significant mediating effect for the impact of IC on total factor productivity. Hypothesis 3 is verified.

With reference to Li and Zhao (2022) [11], the non-parametric percentile bootstrap (1000) method for deviation correction is adopted. Further, the confidence interval of θ_1_×δ_2_ with 95% confidence is estimated to be [0.159, 0.240], where, θ_1_×δ_2_ is the product of the effects of IC on CAUT, and CAUT on LnTFP_LP. Approximately, Sobel test shows that the mediating effect size is 35.23%. These results support the mechanism that effective IC improves capacity utilization, and then increases total factor productivity. In the control variables, the conclusions on L.TAT, L.ROA, L.TQ and L.LnASSET are consistent with those from Model 2. Besides, that on ShrZ is negative and significant (-0.064, *p*< 0.05). Possibly, major shareholders’ “hollowing out” has an adverse impact on total factor productivity. The coefficients on the remaining control variables are not statistically significant.

## 7. Robustness rest

### 7.1 Re-measuring total factor productivity

For the estimation of total factor productivity, both fixed-effects and Olley-Pakes (OP) methods correct simultaneity bias (Olley and Pakes, 1996; Lu and Lian, 2012) [36, 44]. Therefore, with reference to Olley and Pakes (1996) [44], Lu and Lian (2012) [36], and Cheng et al. (2024) [45], the fixed-effects and OP methods are adopted to estimate total factor productivity again. Specifically, Model 5 is constructed to estimate the total factor productivity based on fixed-effects method (TFP_Fe). Model 6 is constructed to calculate that based on OP method (TFP_OP). In Model 5 or 6, LnY_i,t_, LnK_i,t_, LnL_i,t_, and LnM_i,t_ have the same meanings as in Model 1. And YEAR, Region, and IND indicate annual, regional and industry effects, respectively. In Model 6, Age, and SOE represent corporate age, and attribute, respectively. Export is a dummy variable, which is 1, if the enterprise is involved in exporting, and 0 otherwise.

Model 5.


LnYi,t=λ0+λ1LnKi,t+λ2LnLi,t+λ3LnMi,t+λ4∑tYEAR+λ5∑tRegion+λ6∑tIND+εi,t (7)

**Model 6.**

LnYi,t=ω0+ω1LnKi,t+ω2LnLi,t+ω3Agei,t+ω4SOEi,t+ω5Exporti,t+ω6∑tYEAR+ω7∑tRegion+ω8∑tIND+εi,t (8)

For Model 5, the fixed-effects regression is conducted, the coefficients on LnK_i,t_, and LnL_i,t_ are substituted into Eq (9), to obtain TFP_Fe. For Model 6, OP method is adopted. The state variables are Age and LnK. The proxy variable is corporate investment, measured by the cash paid for acquiring fixed assets, intangible assets and other long-term assets. And LnL, YEAR, Region and IND are the free variables. SOE and Export are the control variables. The exit variable is measured according to whether corporate abbreviation and industry changes simultaneously. If the changes occur simultaneously, the value is 1; otherwise, it is 0. Then, those on LnK_i,t_ and LnL_i,t_ are substituted into Eq (10), and TFP_OP can be obtained. Further, TFP_Fe and TFP_OP are used as the explained variables in Models 2 and 4, respectively. For Models 2 and 4, Table 5 reports the results after re-measuring total factor productivity.


$$TFP\_Fe_{i,t}=LnY_{i,t}‐{\hat{\lambda }}_{1}LnK_{i,t}‐{\hat{\lambda }}_{2}LnL_{i,t}$$ (9)

$$TFP\_OP_{i,t}=LnY_{i,t}‐{\hat{\omega }}_{1}LnK_{i,t}‐{\hat{\omega }}_{2}LnL_{i,t}$$ (10)

**Table 5:** Results after re-measuring total factor productivity for Models 2 and 4.

Variable	TFP_Fe		TFP_OP	
	(1)	(2)	(3)	(4)
	Model 2	Model 4	Model 2	Model 4
	Coef. (S.E.)	Coef. (S.E.)	Coef. (S.E.)	Coef. (S.E.)
Intercept	-3.921*** (0.322)	-7.857*** (0.274)	-0.901*** (0.278)	-5.326*** (0.196)
IC	0.555*** (0.033)	0.338*** (0.023)	0.462*** (0.031)	0.218*** (0.016)
CAUT		0.473*** (0.010)		0.532*** (0.008)
L.LEV	0.136*** (0.040)	-0.003 (0.030)	0.117*** (0.038)	-0.040* (0.024)
L.TAT	0.703*** (0.024)	0.316*** (0.021)	0.595*** (0.021)	0.160*** (0.017)
L.ROA	0.317*** (0.077)	0.305*** (0.056)	0.317*** (0.073)	0.305*** (0.043)
L.TQ	0.042*** (0.004)	0.041*** (0.003)	0.023*** (0.003)	0.021*** (0.002)
L.R&D	-0.092*** (0.020)	0.007 (0.014)	-0.134*** (0.018)	-0.023** (0.010)
ShrZ	-0.052 (0.043)	-0.093*** (0.032)	0.007 (0.041)	-0.040 (0.025)
L.ManaHold	0.037 (0.040)	0.036 (0.030)	0.010 (0.045)	0.009 (0.030)
L.LnSALARY	0.002 (0.009)	0.002 (0.007)	-0.005 (0.009)	-0.005 (0.006)
L.LnASSET	0.653*** (0.014)	0.702*** (0.011)	0.323*** (0.012)	0.377*** (0.008)
SOE	-0.043* (0.022)	0.009 (0.016)	-0.076*** (0.025)	-0.017 (0.016)
YEAR/IND/Firm	YES	YES	YES	YES
# of obs.	18727	18727	18727	18727
Within_R^2^	0.699	0.828	0.568	0.837
F_Value	991.94***	2006.17***	561.88***	2143.62***

Note: *** Significant at 1%

** Significant at 5%

* Significant at 10%.

() denotes robust standard errors clustered at corporate level.

From columns 1 to 4, those on IC are positive and significant (0.555, *p*< 0.01; 0.338, *p*< 0.01; 0.462, *p*< 0.01; 0.218, *p*< 0.01). Effective IC has a significant promoting effect on total factor productivity. Again, Hypothesis 1 is verified. In columns 2 and 4, those on CAUT are positive and significant (0.473, *p*< 0.01; 0.532, *p*< 0.01). Capacity utilization reflects organizational efficiency. Sufficient capacity utilization increases total factor productivity. In accordance with those on IC and CAUT, and that on IC in Model 3 from Table 4, it is shown that the mechanism that effective IC strengthens capacity utilization, and increases total factor productivity. Again, Hypothesis 3 is verified. Effective IC is a series of dynamic management activities that continuously evolve [30], which enable enterprises to allocate resources reasonably, to ensure efficient capacity utilization, thereby increasing total factor productivity.

For the control variables, the conclusions on L.TAT, L.ROA, L.TQ, L.R&D, ShrZ, L.LnASSET, and SOE are consistent with those from Table 4. Besides, in columns 1 and 3, the conclusion on L.LEV is consistent with that from Table 4. However, in column 4, that on L.LEV is negative and significant (-0.040, *p*< 0.10), different from that from Table 4. Perhaps, excessive debt pressure increases operational burden, hampering total factor productivity improvement.

### 7.2 Instrumental variable method

There may be reverse causality between IC and capacity utilization, and total factor productivity, resulting in the endogeneity. Therefore, Instrumental variable method is adopted. Appropriate instrumental variables are highly correlated with the endogenous variable, but not with explained variables. With reference to Li and Zhao (2022) [11], Luo et al. (2021) [46], the mean of IC based on industry-region-annual standard (MIC), and whether the enterprise acquires a standard audit report (AUDIT) are adopted as the instrumental variables. In the current year, if the standard audit report is issued, AUDIT is 1; otherwise, it is 0. Based on “peer effect,” the average IC effectiveness of enterprises within same industry and region affect IC effectiveness of this enterprise. And auditor governance promotes IC construction. However, as a higher-dimension variable, MIC has a lower correlation with corporate capacity utilization, and total factor productivity. Moreover, it is difficult for audit governance to directly affect capacity utilization, and total factor productivity. Therefore, MIC and AUDIT meet the relevance and exclusivity as the instrumental variables. Further, with reference to Li et al. (2022) [47], Two Stage Least Square (2SLS) is adopted for analyses. In the first stage, the first-order lag of MIC (L.MIC), and that of AUDIT (L.AUDIT) are considered, to examine the impacts of IC on capacity utilization, and total factor productivity, again.

Table 6 reports the results with 2SLS regression. At the bottom, the coefficients on L.MIC and L.AUDIT are the first-stage results. From columns 1 to 3, those on L.MIC are positive and significant (0.235, *p*< 0.01; 0.235, *p*< 0.01; 0.234, *p*< 0.01); and as are those on L.AUDIT (0.134, *p*< 0.01; 0.134, *p*< 0.01; 0.133, *p*< 0.01), implying that the “peer effect” within same industry and region, and audit governance facilitate IC effectiveness. Meanwhile, in accordance with Kleibergen-Paap rk LM statistic (284.232, *p* = 0.000; 284.232, *p* = 0.000; 280.975, *p* = 0.000), Kleibergen-Paap rk Wald F statistic (234.687> 19.930; 234.687> 19.930; 231.144> 19.930), there are no under-identification, and weak identification; and Hansen J statistic (1.153, *p*> 0.10; 0.967, *p*> 0.10; 0.328, *p*> 0.10), indicating that the hypothesis that both the instrumental variables are exogenous cannot be rejected at 10% significance. These results indicate that the instrumental variables are reasonable.

**Table 6:** Test results for instrumental variable method.

Variable	(1)	(2)	(3)
	Model 2	Model 3	Model 4
	Coef. (S.E.)	Coef. (S.E.)	Coef. (S.E.)
Intercept	-6.845*** (0.092)	7.080*** (0.137)	-10.095*** (0.069)
IC	0.675*** (0.101)	0.449*** (0.160)	0.468*** (0.069)
CAUT			0.459*** (0.005)
L.LEV	0.196*** (0.021)	0.251*** (0.031)	0.080*** (0.014)
L.TAT	1.100*** (0.012)	1.326*** (0.016)	0.492*** (0.011)
L.ROA	0.279*** (0.065)	0.151 (0.095)	0.210*** (0.045)
L.TQ	0.028*** (0.003)	0.004 (0.004)	0.027*** (0.002)
L.R&D	-0.068*** (0.008)	-0.227*** (0.011)	0.037*** (0.005)
ShrZ	-0.003 (0.024)	0.069* (0.038)	-0.034** (0.017)
L.ManaHold	0.088*** (0.014)	0.023 (0.021)	0.077*** (0.010)
L.LnSALARY	0.040*** (0.005)	0.045*** (0.007)	0.020*** (0.003)
L.LnASSET	0.640*** (0.004)	-0.092*** (0.005)	0.682*** (0.003)
SOE	-0.011 (0.007)	0.001 (0.010)	-0.011** (0.005)
YEAR/IND/Firm	YES	YES	YES
# of obs.	18727	18727	18727
F_Value	3004.62***	415.01***	5660.28***
L.MIC	0.235*** (0.014)	0.235*** (0.014)	0.234*** (0.014)
L.AUDIT	0.134*** (0.012)	0.134*** (0.012)	0.133*** (0.012)
Kleibergen-Paap rk LM statistic	284.232 [0.000]	284.232 [0.000]	280.975 [0.000]
Kleibergen-Paap rk Wald F statistic	234.687 {19.930}	234.687 {19.930}	231.144 {19.930}
Hansen J statistic	1.153 [0.283]	0.967 [0.325]	0.328 [0.567]

Note: *** Significant at 1%

** Significant at 5%

* Significant at 10%.

() denotes robust standard errors clustered at corporate level.

{} denotes Stock-Yogo weak ID test critical value at 10%.

[] denotes *p*-values.

In the second stage, from columns 1 to 3, those on IC are positive and significant (0.675, *p*< 0.01; 0.449, *p*< 0.01; 0.468, *p*< 0.01). Effective IC forms a good supervisory and incentive mechanism, improves decision-making efficiency, optimizes market feedback, enhances total factor productivity and capacity utilization. In column 3, that on CAUT is positive and significant (0.459, *p*< 0.01). Adequate capacity utilization improves total factor productivity. Combining those on IC from Models 2 to 4, and that on CAUT in Model 4, this study argues that good internal governance reduces overcapacity, improves capacity utilization, and enhances total factor productivity. Approximately, the mediating effect size of capacity utilization for the impact of IC on total factor productivity is 30.53% (θ_1_×δ_2_/β_1_ = 0.449×0.459 /0.675). Again, Hypotheses 1 to 3 are verified.

For the control variables, in Model 2 or 4, the conclusions on L.LEV, L.TAT, L.ROA, L.TQ, ShrZ, L.LnASSET, and SOE are consistent with those from Table 4. In column 1, the conclusion on L.R&D is consistent with that from Table 4. However, in column 3, that on L.R&D is positive and significant (0.037, *p*< 0.01), different from that from Table 4. Technological innovation is an important support for enhancing total factor productivity [48]. Besides, in columns 1 and 3, those on L.ManaHold are positive and significant (0.088, *p*< 0.01; 0.077, *p*< 0.01); as are those on L.LnSALARY (0.040, *p*< 0.01; 0.020, *p*< 0.01). Good equity and compensation incentives motivate executives to perform duties diligently, to increase total factor productivity. In Model 3, the conclusions on L.LEV, L.TAT, L.R&D, and L.LnASSET are consistent with those from Table 4. Besides, those on ShrZ, and L.LnSALARY are positive and significant (0.069, *p*< 0.10; 0.045, *p*< 0.01). Major shareholder governance and executive compensation incentives stimulate operational potential, thereby improving capacity utilization.

### 7.3 Heckman two-step tests

Effective IC is an important guarantee for achieving strategic objectives. However, the governance and the management may selectively allocate resources in IC construction. The management may override IC, resulting in IC failure. Therefore, enterprises have some degree of “self-selection” in IC construction [11]. To alleviate the endogeneity caused by the “self-selection” problem, Heckman two-step tests are adopted for analyses. In the first step, the Probit Model 7 on IC effectiveness is constructed, to estimate the inverse Mills ratio (Imills). A dummy variable on IC effectiveness (DIC) is set. Relative to the previous year, in the current one, if IC effectiveness shows improvement, DIC is 1; otherwise, DIC is 0, indicating that IC effectiveness remains unchanged or decreases. Meanwhile, in line with Li and Zhao (2022) [11], the first-order lags of MIC and AUDIT are added as the instrumental variables, to meet Exclusion restriction. MIC and AUDIT have the same meanings as in Section 7.2. In the second step, the Imills is introduced into the original Models 2 to 4 as a control variable, respectively, to examine the impacts of IC on capacity utilization, and total factor productivity.

**Model 7.**

$$\begin{array}{l} DIC_{i,t}=\zeta_{0}+\zeta_{1}LEV_{i,t‐1}+\zeta_{2}TAT_{i,t‐1}+\zeta_{3}ROA_{i,t‐1}+\zeta_{4}TQ_{i,t‐1}+\zeta_{5}R\&D_{i,t‐1}+\zeta_{6}ShrZ_{i,t}+\\ \;\zeta_{7}ManaHold_{i,t‐1}+\zeta_{8}LnSALARY_{i,t‐1}+\zeta_{9}LnASSET_{i,t‐1}+\zeta_{10}SOE_{i,t}+\\ \;\zeta_{11}MIC_{i,t‐1}+\zeta_{12}AUDIT_{i,t‐1}+\zeta_{13}\sum_{t}YEAR+\zeta_{14}\sum_{t}IND+\varepsilon_{i,t} \end{array}$$ (11)

In Table 7, for the first-step regression, the coefficients on L.MIC and L.AUDIT are statistically significant (-6.245, *p*< 0.01; 0.406, *p*< 0.01), implying that the variables introduced into the sample selection regression are valid. In the second step, from columns 1 to 2, those on Imills are statistically significant (0.035, *p*< 0.10; 0.067, *p*< 0.05), indicating that the “self-selection” exists in the observations. Heckman two-step model can mitigate the regression bias caused by the “self-selection” problem. From Models 2 to 4, those on IC are positive and significant (0.861, *p*< 0.01; 0.496, *p*< 0.01; 0.631, *p*< 0.01), indicating again that effective IC impacts total factor productivity and capacity utilization positively. In Model 4, that on CAUT is positive and significant (0.463, *p*< 0.01). Lower idle capacity helps increase total factor productivity. In accordance with those on IC, and CAUT, the improvement of capacity utilization has a significant mediating effect for the impact of IC on total factor productivity.

**Table 7:** Results for Heckman two-step tests.

Variable	(1)	(2)	(3)
	Model 2	Model 3	Model 4
	Coef. (S.E.)	Coef. (S.E.)	Coef. (S.E.)
Intercept	-6.796*** (0.113)	7.207*** (0.166)	-10.133*** (0.091)
IC	0.861***(0.065)	0.496*** (0.096)	0.631*** (0.048)
CAUT			0.463*** (0.005)
L.LEV	0.185*** (0.027)	0.269*** (0.040)	0.061*** (0.020)
L.TAT	1.123*** (0.012)	1.343*** (0.017)	0.501*** (0.011)
L.ROA	0.235*** (0.078)	0.072 (0.114)	0.202*** (0.057)
L.TQ	0.028*** (0.004)	0.001 (0.005)	0.028*** (0.003)
L.R&D	-0.075*** (0.009)	-0.233*** (0.014)	0.033*** (0.007)
ShrZ	0.001 (0.035)	0.078 (0.051)	-0.035 (0.025)
L.ManaHold	0.114*** (0.022)	0.064** (0.032)	0.085*** (0.016)
L.LnSALARY	0.042*** (0.007)	0.055*** (0.010)	0.016*** (0.005)
L.LnASSET	0.631*** (0.005)	-0.101*** (0.007)	0.678*** (0.004)
SOE	-0.010 (0.010)	0.001 (0.014)	-0.011 (0.007)
Imills	0.035* (0.019)	0.067** (0.027)	0.004 (0.014)
YEAR/IND/Firm	YES	YES	YES
# of obs.	18727	18727	18727
L.MIC	-6.245*** (0.178)	-6.245*** (0.178)	-6.245*** (0.178)
L.AUDIT	0.406*** (0.085)	0.406*** (0.085)	0.406*** (0.085)
Wald_chi^2^	66081.31***	11982.41***	130996.46***

Note: *** Significant at 1%

** Significant at 5%

* Significant at 10%.

() denotes standard errors.

In terms of the control variables, in Models 2 and 4, the conclusions on L.LEV, L.TAT, L.ROA, L.TQ, L.R&D, L.ManaHold, L.LnSALARY, and L.LnASSET are consistent with those from Table 4, or 6. In Model 3, the conclusions on L.LEV, L.TAT, L.R&D, L.LnSALARY, and L.LnASSET are consistent with those from Table 4, or 6; besides, that on L.ManaHold is positive and significant (0.064, *p*< 0.05). Appropriate equity incentives motivate the management to perform duties diligently, fully perceive changes in external economic conditions, rationally arrange production capacity, optimize capacity structure, and improve capacity utilization, to effectively prevent operational risks.

### 7.4 Grouping test for Model 2, and constructing Model 8

To further verify Hypothesis 3 above, referring to the research of Li (2022) [49], based on industry-annual standard, this study estimates the median of CAUT, denoted as CAUT50. A dummy variable is set, and expressed as DCAUT. In the current year, If CAUT is greater than CAUT50, DCAUT is 1, indicating higher capacity utilization. Otherwise, DCAUT is 0, indicating lower capacity utilization. Further, with DCAUT = 1 and DCAUT = 0, group regression is performed on Model 2. In Table 8, columns 1 and 2 report the results with DCAUT = 1, and DCAUT = 0, respectively. Meanwhile, with reference to Li and Liu (2021) [50], the following Model 8 with an interaction term (IC×DCAUT) is constructed, to further examine the reliability of Hypothesis 3. To alleviate the multicollinearity, in accordance with Balli and Sørensen (2013) [51], in the interaction term, IC is de-averaged by industry and year. In Table 8, column 3 reports the results for Model 8.

**Table 8:** Results for grouping test for Model 2, and those for Model 8.

Variable	Model 2		Model 8
	(1)	(2)	(3)
	DCAUT = 1	DCAUT = 0	
	Coef. (S.E.)	Coef. (S.E.)	Coef. (S.E.)
Intercept	-1.569*** (0.414)	-3.762*** (0.466)	-3.384*** (0.280)
IC	0.406*** (0.039)	0.438*** (0.039)	0.533*** (0.039)
DCAUT			0.256*** (0.008)
IC×DCAUT			-0.164*** (0.052)
L.LEV	0.117** (0.056)	-0.003 (0.055)	0.059 (0.036)
L.TAT	0.434*** (0.027)	0.792*** (0.064)	0.594*** (0.021)
L.ROA	0.238** (0.103)	0.241** (0.101)	0.313*** (0.069)
L.TQ	0.030*** (0.004)	0.035*** (0.005)	0.035*** (0.003)
L.R&D	-0.059** (0.030)	-0.037* (0.021)	-0.084*** (0.017)
ShrZ	-0.010 (0.044)	-0.068 (0.057)	-0.029 (0.037)
L.ManaHold	-0.126** (0.052)	0.138*** (0.050)	0.003 (0.040)
L.LnSALARY	0.013 (0.011)	-0.001 (0.014)	0.002 (0.009)
L.LnASSET	0.460*** (0.018)	0.529*** (0.021)	0.517*** (0.012)
SOE	-0.010 (0.036)	-0.038 (0.025)	-0.040* (0.021)
YEAR/IND/Firm	YES	YES	YES
# of obs.	9189	9538	18727
Within_R^2^	0.680	0.609	0.679
F_Value	429.11***	311.46***	859.37***

*** Significant at 1%

** Significant at 5%

* Significant at 10%.

() denotes robust standard errors clustered at corporate level.

**Model 8.**

$$\begin{array}{l} LnTFP\_LP_{i,t}=\kappa_{0}+\kappa_{1}IC_{i,t}+\kappa_{2}CAUT_{i,t}+\kappa_{3}IC_{i,t}\times DCAUT_{i,t}+\kappa_{4}LEV_{i,t‐1}+\kappa_{5}TAT_{i,t‐1}+\\ \;\kappa_{6}ROA_{i,t‐1}+\kappa_{7}TQ_{i,t‐1}+\kappa_{8}R\&D_{i,t‐1}+\kappa_{9}ShrZ_{i,t}+\kappa_{10}ManaHold_{i,t‐1}+\\ \;\kappa_{11}LnSALARY_{i,t‐1}+\kappa_{12}LnASSET_{i,t‐1}+\kappa_{13}SOE_{i,t}+\kappa_{14}\sum_{t}YEAR+\\ \;\kappa_{15}\sum_{t}IND+\varepsilon_{i,t} \end{array}$$ (12)

From columns 1 to 2, those coefficients on IC are positive and significant (0.406, *p*< 0.01; 0.438, *p*< 0.01). And Fisher’s permutation test shows that the differences in those on IC are negative and significant (-0.136, *p*< 0.01). In column 3, that on IC×DCAUT is negative and significant (-0.164, *p*< 0.01). The above results show that, with lower capacity utilization, effective IC enhances corporate total factor productivity more significantly. Effective IC alleviates insufficient capacity utilization, facilitates capacity utilization efficiency, and then enhances total factor productivity. Again, Hypothesis 3 above is verified.

For the control variables, in column 1, 2, or 3, the conclusions on L.LEV, L.TAT, L.ROA, L.TQ, L.R&D, L.LnASSET, and SOE are consistent with those from Table 4. Besides, in column 2, that on L.ManaHold is positive and significant (0.138, *p*< 0.01), which tends to be consistent with those from Table 6. However, in column 1, that on L.ManaHold is negative and significant (-0.126, *p*< 0.05), different from that from Table 6. Perhaps, higher managerial ownership leads to insider control, and managers prioritize own interests over corporate overall interests, affecting total factor productivity adversely.

## 8. Heterogeneity discussion

In the context of current economic development, the environmental uncertainty faced by enterprises is becoming increasingly apparent. Environmental uncertainty refers to the unpredictability of changes in various elements in the business environment [52]. Environmental uncertainty is an important factor affecting corporate decisions [14]. It is an important challenge that enterprises have to face in operation. Then, under differentiated environmental uncertainty, is there heterogeneity in the impacts of IC on total factor productivity, and capacity utilization, respectively? Is the mechanism that effective IC improves capacity utilization, and increases total factor productivity heterogeneous?

With reference to Ghosh and Olsen (2009) [53], Shen et al. (2012) [54], and Wang et al. (2023) [12], the variation of sales revenue adjusted based on industry-annual criterion is adopted to measure environmental uncertainty. First, the following Model 9 is constructed, and the data of each enterprise over past 5 years is used for OLS regression, and the residuals represent abnormal sales revenue. In Model 9, Sale represents sales revenue, and YEAR means annual variable. The data from the current year to previous 4 years are adopted, and YEAR is assigned values from 5 to 1. Second, the standard deviation of abnormal sales revenue for 5 years is divided by the average sales revenue for the same period, to obtain the unadjusted environmental uncertainty (EU). Third, based on industry-annual standard, the median of EU is estimated, and expressed as MEU. The ratio of EU to MEU is used as the industry-adjusted environmental uncertainty (EnvU). According to industry-annual criterion, the median of EnvU is estimated, and expressed as *M*_*0*_. Further, a dummy variable (DEU) is set. With EnvU> *M*_*0*_, DEU is 1, indicating a higher environmental uncertainty. Otherwise, DEU is 0, indicating a lower environmental uncertainty. Based on the first-order lag of DEU (L.DEU), L.DEU = 0 and L.DEU = 1 are distinguished, and grouping analyses are conducted from Models 2 to 4. Table 9 reports the results for Heterogeneity discussion.

**Table 9:** Results for Heterogeneity discussion.

Variable	L.DEU = 0			L.DEU = 1		
	(1)	(2)	(3)	(4)	(5)	(6)
	Model 2	Model 3	Model 4	Model 2	Model 3	Model 4
	Coef. (S.E.)	Coef. (S.E.)	Coef. (S.E.)	Coef. (S.E.)	Coef. (S.E.)	Coef. (S.E.)
Intercept	-3.030*** (0.485)	8.134*** (0.636)	-6.696*** (0.450)	-3.142*** (0.532)	7.061*** (0.630)	-6.816*** (0.447)
IC	0.364*** (0.045)	0.334*** (0.063)	0.213*** (0.030)	0.522*** (0.053)	0.483*** (0.074)	0.271*** (0.033)
CAUT			0.451*** (0.015)			0.520*** (0.014)
L.LEV	0.163*** (0.052)	0.259*** (0.090)	0.047 (0.040)	0.053 (0.077)	0.395*** (0.109)	-0.153*** (0.055)
L.TAT	0.726*** (0.035)	0.834*** (0.055)	0.350*** (0.029)	0.623*** (0.035)	0.717*** (0.048)	0.250*** (0.028)
L.ROA	0.384*** (0.104)	0.138 (0.173)	0.321*** (0.068)	0.123 (0.114)	-0.042 (0.155)	0.145* (0.083)
L.TQ	0.025*** (0.004)	0.001 (0.007)	0.025*** (0.003)	0.049*** (0.008)	0.021* (0.011)	0.039*** (0.006)
L.R&D	-0.060** (0.026)	-0.087** (0.041)	-0.021 (0.019)	-0.141*** (0.030)	-0.258*** (0.042)	-0.006 (0.018)
ShrZ	-0.068 (0.042)	0.062 (0.062)	-0.096*** (0.034)	-0.116 (0.094)	-0.004 (0.120)	-0.115* (0.065)
L.ManaHold	-0.074 (0.060)	-0.146 (0.101)	-0.008 (0.047)	0.095 (0.092)	0.182 (0.134)	0.001 (0.073)
L.LnSALARY	-0.005 (0.012)	-0.016 (0.021)	0.003 (0.009)	-0.007 (0.018)	-0.019 (0.024)	0.003 (0.012)
L.LnASSET	0.502*** (0.020)	-0.089*** (0.031)	0.543*** (0.016)	0.520*** (0.024)	-0.041 (0.029)	0.541*** (0.019)
SOE	-0.067** (0.033)	-0.144*** (0.049)	-0.002 (0.023)	-0.080** (0.040)	-0.133** (0.059)	-0.011 (0.024)
YEAR/IND/Firm	YES	YES	YES	YES	YES	YES
# of obs.	7267	7267	7267	6822	6822	6822
Within_R^2^	0.639	0.229	0.801	0.580	0.245	0.807
F_Value	278.48***	46.73***	615.46***	186.38***	43.91***	547.17***

Note: *** Significant at 1%

** Significant at 5%

* Significant at 10%.

() denotes robust standard errors clustered at corporate level.

**Model 9.**

$$Sale_{i,t}=\varphi_{0}+\varphi_{1}\sum_{t}YEAR+\varepsilon_{i,t}$$ (13)

In Table 9, columns 1 to 3, and columns 4 to 6 report the results with L.DEU = 0, and L.DEU = 1, respectively. From columns 1 to 3, those on IC are positive and significant (0.364, *p*< 0.01; 0.334, *p*< 0.01; 0.213, *p*< 0.01); as are those on IC (0.522, *p*< 0.01; 0.483, *p*< 0.01; 0.271, *p*< 0.01) from columns 4 to 6. With higher or lower environmental uncertainty, effective IC improves total factor productivity and capacity utilization. With L.DEU = 0 and L.DEU = 1, from Models 2 to 4, Fisher’s permutation tests show that the differences in those on IC are negative and significant (-0.216, *p*< 0.01; -0.147, *p*< 0.10; -0.120, *p*< 0.01). With higher environmental uncertainty, effective IC improves capacity utilization to a greater extent; and effective IC has a more significant marginal promoting effect on total factor productivity, differing from that Wang et al. (2023) [12] believed that environmental uncertainty negatively moderated the impact of IC on total factor productivity. Compared with lower environmental uncertainty, under higher environmental uncertainty, the governance and the management respond cautiously, transform pressure into motivation, and strengthen IC construction, to ensure sustainable and healthy operation, cope with larger fluctuations in market demands, promote efficiency and power changes, and enhance total factor productivity and capacity utilization.

In columns 3 and 6, those on CAUT are positive and significant (0.451, *p*< 0.01; 0.520, *p*< 0.01). Sufficient capacity utilization promotes total factor productivity. With L.DEU = 0 and L.DEU = 1, in Model 4, the difference in those on CAUT is negative and significant (-0.071, *p*< 0.01). With higher environmental uncertainty, the improvement in capacity utilization has a stronger promoting effect on total factor productivity. Along with production factors invested in operation, enterprises acquire higher marginal returns, to cope with higher environmental uncertainty. In accordance with those on IC from Models 2 to 4, and those on CAUT in Model 4, it is argued that greater capacity utilization has a significant mediating effect for the impact of IC on total factor productivity, under both lower and higher environmental uncertainties.

With L.DEU = 0 and L.DEU = 1, Sobel tests show that the mediating effect sizes are 34.53%, and 38.02%, respectively. Therefore, with higher environmental uncertainty, it is shown that the greater mediating effect of capacity utilization for the impact of IC on total factor productivity. With unstable market demands, the governance and the management refine relevant policies and procedures, to ensure IC effectiveness. Effective IC shows a more pronounced marginal effect, ensuring efficient resource allocation, significantly improving capacity utilization, enhancing supply capacity and value-added products, and increasing total factor productivity.

For the control variables, in Model 2 or 4, the conclusions on L.LEV, L.TAT, L.ROA, L.TQ, L.R&D, ShrZ, L.LnASSET, and SOE are consistent with those from Table 4, or 5. In Model 3, the conclusions on L.LEV, L.TAT, L.R&D, L.LnASSET, and SOE are consistent with those from Table 4. Besides, in column 5, that on L.TQ is positive and significant (0.021, *p*< 0.10). Good development motivates the management to improve capacity utilization, to protect stakeholders’ interests.

## 9. Conclusion and recommendation

### 9.1 Conclusion

Based on IC theory and Principal-agent theory, this study explores the impacts of IC on total factor productivity and capacity utilization, and elucidates the theoretical mechanism among effective IC, capacity utilization, and total factor productivity. The results show that effective IC promotes quality, efficiency and power changes, and enhances total factor productivity and capacity utilization. Sufficient capacity utilization has a mediating effect for the impact of IC on total factor productivity. In Robustness test, the conclusions are verified again, by re-measuring total factor productivity, Instrumental variable method, Heckman two-step tests, Grouping test and constructing Model with a interaction term.

Heterogeneity discussion shows that compared with lower environmental uncertainty, with higher environmental uncertainty, effective IC has a more significant marginal promoting effect on total factor productivity and capacity utilization, differing from that Wang et al. (2023) [12] argued that environmental uncertainty negatively moderated the effect of IC on total factor productivity. Moreover, with higher environmental uncertainty, sufficient capacity utilization has a greater mediating effect for the impact of IC on total factor productivity, to cope with larger fluctuations in market demands. The study enriches the relevant literature that IC enables corporate operation, which has certain significance for enhancing IC effectiveness, coping with adverse events, improving operation efficiency and quality, and shaping competitive advantages.

### 9.2 Recommendation

More attention has been paid to improving the quality and efficiency of economic development. Aligned with high-quality economic development, the “proactive government” and “effective markets” are combined, and efforts are made to build a management system with effective market mechanisms, dynamic micro-entities, and appropriate macro-controls, to provide more material guarantee and hardware support for increasing total factor productivity. The regulators guide enterprises to strengthen IC construction and enhance IC effectiveness, continuously optimize resource allocation, reduce overcapacity, create a good foundation for improving capacity utilization and total factor productivity, promote operational quality, efficiency and power transformation, and obtain investment returns while achieving sustainable development, to ensure higher-quality development.

Enterprises adhere to the sustainable development strategy, actively develop and refine relevant policies and procedures, enhance IC effectiveness, mitigate opportunism and moral hazard, optimize resource allocation, increase output efficiency, reduce operational risks, to improve capacity utilization, and thereby enhance total factor productivity. The mechanism that effective IC improves capacity utilization, and increases total factor productivity is promoted, to achieve transformation, upgrading and benign development. As environmental uncertainty increases, enterprises focus more on improving IC system, comprehensively coping with higher operational risks, utilizing resources reasonably and efficiently, integrating resources and facilities, to ensure capacity utilization growth, while improving capacity allocation, synergistically increase total factor productivity.

### 9.3 Limitation and prospect

Effective IC alleviates agency conflicts, and improves operational efficiency [26]. Due to space limitations, this study only explores the mechanism among IC, capacity utilization, and total factor productivity. Nevertheless, effective IC may affect total factor productivity in other ways. In future studies, it is worth exploring the other mechanisms that effective IC enables total factor productivity. Meanwhile, based on differentiated environmental uncertainty, this study explores the heterogeneous mechanism that IC improves capacity utilization, and increases total factor productivity. Besides, economic policy uncertainty has a moderating effect for the impact of IC on total factor productivity [55]. Therefore, according to differentiated economic policy uncertainty, it is worth investigating the possible heterogeneous mechanism among IC, capacity utilization, and total factor productivity, to provide more empirical evidence for acquiring competitive advantages.

## Supporting information

S1 Dataset The data set used in this article for discussion and analysis. (ZIP)
